# Tratamento cirúrgico da instabilidade da sínfise púbica com enxertos de tendão semitendíneo: Relato de dois casos com descrição de técnica cirúrgica

**DOI:** 10.1055/s-0046-1820488

**Published:** 2026-06-16

**Authors:** Henrique Antônio Berwanger de Amorim Cabrita, Tatiana Ciocler Trahtenberg Guttmann, Adriano Rodrigues da Silva

**Affiliations:** 1 Instituto de Ortopedia e Traumatologia, Hospital das Clínicas, Faculdade de Medicina, Universidade de São Paulo, São Paulo, SP, Brasil; 2 Departamento de Ortopedia e Traumatologia, Hospital Israelita Albert Einstein, São Paulo, SP, Brasil

**Keywords:** instabilidade articular, osso púbico, osteíte, sínfise púbica, tendões dos músculos isquiotibiais, hamstring tendons, joint instability, osteitis, pubic bone, symphysis pubis

## Abstract

A sínfise púbica é uma articulação fundamental para a estabilidade do anel pélvico. A instabilidade desta articulação pode causar dor crônica e limitação funcional significativa, especialmente em atletas e indivíduos jovens. Embora o tratamento conservador seja a primeira linha de manejo, casos refratários podem exigir abordagem cirúrgica. Este estudo propõe apresentar uma descrição de técnica cirúrgica utilizada em dois pacientes atletas de futebol que foram submetidos à reconstrução da sínfise púbica com via de acesso de Pffanestiel, passagem de enxerto autólogo do 'tendão semitendinoso através de túneis ósseos nos ossos do púbis, configurados em oito e fixados com fios de sutura inabsorvíveis. A técnica visou preservar a mobilidade fisiológica da sínfise púbica. Após 6 meses, houve melhora de mais de 30 pontos do Escore de Harris Modificado para o Quadril e retorno funcional satisfatório às atividades físicas com retorno ao esporte após 6 e 8 meses, respectivamente. Houve melhora da estabilidade pélvica radiológica durante o seguimento de 5 anos. A técnica demonstrou ser segura, com baixa morbidade e bons resultados clínicos, permitindo a preservação da mobilidade pélvica e o retorno ao esporte. Apesar do baixo número de casos, esta alternativa cirúrgica é reprodutível, com vantagens biomecânicas em comparação com a artrodese da sínfise púbica e baixo índice de complicações.

## Abstract (en)

The pubic symphysis is a key joint for the stability of the pelvic ring. Pubic symphysis instability may lead to chronic pain and significant functional limitation, particularly in athletes and young subjects. Although conservative management is the first-line approach, refractory cases may require surgical intervention. The present study aimed to describe a surgical technique used in two soccer players who underwent pubic symphysis reconstruction through a Pfannenstiel approach with an autologous semitendinosus tendon graft passed through bone tunnels in the pubic bones in a figure- eight configuration and fixed with non-absorbable sutures. This technique's goal is to preserve the physiological mobility of the pubic symphysis. At 6 months, both patients showed an improvement of more than 30 points in the modified Harris Hip Score. Moreover, they achieved satisfactory functional recovery, returning to sports at 6 and 8 months, respectively. Radiographic evaluation demonstrated improved pelvic stability over a 5-year follow-up period. The technique proved to be safe, with low morbidity, and good clinical outcomes, allowing pelvic mobility preservation and return to sports. Despite the small number of cases, this surgical approach appears to be reproducible, with biomechanical advantages over pubic symphysis arthrodesis and a low complication rate.

## Introdução

A sínfise púbica, uma articulação que conecta os ossos púbicos, é essencial para a estabilidade do anel pélvico e para a transmissão de forças entre o tronco e os membros inferiores. 1 Sua disfunção pode causar dor, limitação funcional e comprometimento da performance esportiva.

A instabilidade da sínfise púbica pode ter origem traumática, gestacional, por sobrecarga mecânica ou desequilíbrios musculares. Em atletas, particularmente os envolvidos em esportes com mudanças bruscas de direção e corrida de longa distância, microtraumas de repetição podem desencadear instabilidade crônica da sínfise púbica. 2 3

Biomecanicamente, a pelve funciona como um elo entre forças ascendentes e descendentes, com atuação de grupos musculares antagonistas como adutores, abdominais e isquiotibiais. O desequilíbrio entre esses vetores pode gerar forças de cisalhamento e rotação sobre a sínfise púbica, promovendo microinstabilidades, inflamação e, em casos crônicos, degeneração do disco interpubiano. 2

O diagnóstico da instabilidade da sínfise púbica é desafiador. Os sintomas são inespecíficos e podem mimetizar outras causas de dor na região do quadril, como o impacto femoroacetabular e a osteíte púbica. A avaliação clínica, associada a exames de imagem—principalmente a radiografia dinâmica em Flamingo e ressonância magnética—é essencial para o diagnóstico correto. 1 4 5 6

O tratamento conservador é a primeira linha de manejo, envolvendo fisioterapia, analgesia e repouso. Apesar dessas medidas, alguns casos, especialmente aqueles com uma instabilidade óssea radiológica maior que 5 mm, persistem sintomáticos e torna-se necessária a intervenção cirúrgica. 7 A artrodese, embora eficaz na eliminação da instabilidade, pode comprometer a biomecânica pélvica e gerar limitação funcional. 4 Além disso, o índice de complicações pode chegar a 25%, conforme descrito em algumas séries de casos, com artrose da articulação sacro-ilíaca devido à alteração biomecânica da cintura pélvica. 1 Nesse cenário, a reconstrução ligamentar com enxerto autólogo surge como uma alternativa promissora, com potencial de restaurar a estabilidade preservando a mobilidade fisiológica da articulação.

O presente estudo visa apresentar uma técnica cirúrgica inédita para reconstrução da sínfise púbica utilizando enxertos autólogos dos tendões isquiotibiais no tratamento de dois pacientes com diagnóstico clínico e radiológico de instabilidade da sínfise púbica refratária ao tratamento conservador. O estudo foi aprovado pelo Comitê de Ética em Pesquisa (CAAE: n° 90192625.4.0000.0071) e os participantes assinaram um Termo de Consentimento Livre e Esclarecido.

**Fig. 1:**
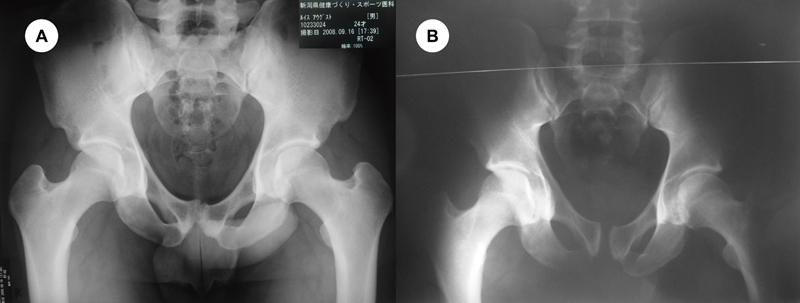
( **A,B** ) Radiografia anteroposterior e *inlet* do quadril demonstrando irregularidade cortical e redução do espaço suprapúbico.

O 1 ^o^ caso foi o de um paciente do sexo masculino, com 26 anos de idade, atleta de futebol de alto rendimento, com dor púbica crônica desde 2007, associada à marcha claudicante e limitação dos quadris. Nos testes para quadril, referiu dor durante manobras de impacto anterior e posterior, além dos testes de Patrick, Grava e Valsalva. Os exames de imagem ( Fig. 1 ) mostraram alterações ósseas justacorticais na superfície da sínfise púbica, e a ressonância revelou lesões capsulares e peritendíneas. Após falha do tratamento conservador, incluindo infiltrações e três cirurgias (tenotomia dos adutores, correção de hérnia inguinal e ressecção de sínfise púbica), foi submetido à reconstrução da sínfise com enxerto autólogo do tendão semitendíneo. Sua radiografia em série de Flamigo apresentava uma discrepância de altura dos ramos púbicos de 25 mm (sendo o normal até 5 mm), o que caracteriza a instabilidade púbica, além de um afastamento de 20 mm dos ossos púbicos. 7 No mesmo tempo cirúrgico, realizou-se artroscopia do quadril direito para reinserção labral e correção de deformidade tipo came. O Escore de Harris Modificado para o Quadril passou de 45 no pré-operatório para 92 em 6 meses e 90 em 5 anos de seguimento. O retorno ao esporte profissional ocorreu após 6 meses de cirurgia.

O 2 ^o^ caso é o de um paciente do sexo masculino, com 22 anos de idade, atleta de futebol profissional, com dor púbica progressiva havia 6 meses. Apresentava testes negativos de dor intra-articular, com dor em sínfise púbica. Os sintomas foram refratários ao tratamento conservador por 6 meses com limitação de desempenho esportivo e Escore de Harris Modificado para o Quadril de 58. Na avaliação radiográfica em posição de flamingo avaliou-se uma diferença de 18 mm em apoios entre o lado direito e esquerdo dos ramos púbicos. Diante da refratariedade clínica, optou-se pela reconstrução da sínfise púbica. O paciente voltou ao futebol profissional após 8 meses de reabilitação, com evolução do escore de Harris modificado para 90 em 6 meses e 97 em 5 anos.

### Técnica cirúrgica

**Fig. 2:**
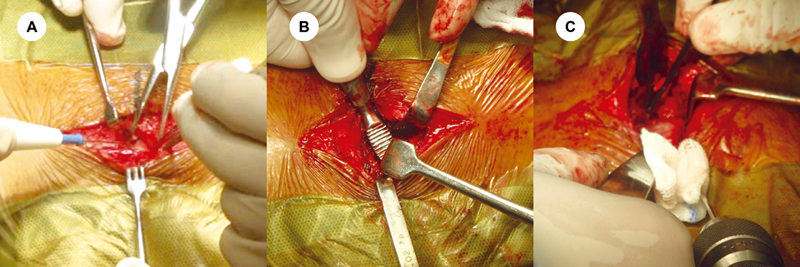
( **A** ) Incisão de Pfannenstiel; ( **B** ) Dissecção por planos e descolamento da musculatura; ( **C** ) Confecção de túneis ósseos nos ramos púbicos bilateralmente, a cerca de 1,5 cm lateral à sínfise púbica, com direção anteroinferior para posterosuperior.

Os pacientes foram submetidos ao procedimento sob anestesia geral, em decúbito dorsal horizontal. Foi realizada incisão transversa suprapúbica do tipo Pfannenstiel, com aproximadamente 5 cm de extensão, a cerca de 2 cm proximal ao tubérculo púbico ( Fig. 2A ). A dissecção por planos foi realizada até a exposição da fáscia anterior do músculo reto abdominal, a qual foi incisada longitudinalmente na linha alba para afastamento lateral das bainhas musculares dos retos abdominais ( Fig. 2B ).

A dissecção cuidadosa da região posterior da sínfise púbica foi realizada manualmente, a fim de evitar lesões à bexiga e ao plexo venoso retro-púbico. Um afastador foi posicionado obliquamente ao tubérculo púbico para otimizar a exposição do aspecto distal dos retos abdominais.

Utilizou-se como enxerto o tendão semitendíneo do membro não dominante do paciente, extraído por um cirurgião de joelho. As extremidades do enxerto foram preparadas com suturas tipo Krakow para facilitar a manipulação e fixação.

**Fig. 3:**
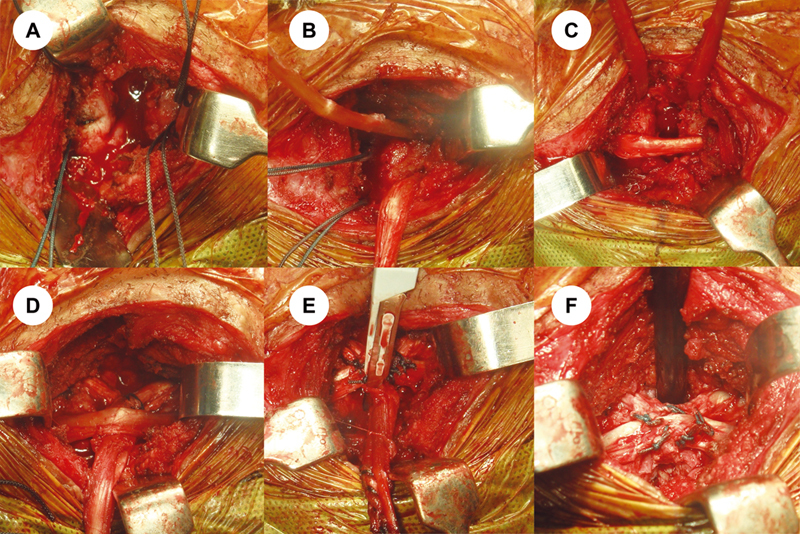
( **A** ) Posicionamento do enxerto de semitendíneo na sínfise púbica; ( **B** ) Passagem do enxerto pelo primeiro túnel ósseo no sentido cranial-caudal; ( **C** ) Configuração em “U” do enxerto pelos túneis; ( **D** ) Tensionamento do enxerto para redução da diástase articular e fixação do enxerto na configuração de “8”; ( **E** ) Ressecção do excesso de enxerto; ( **F** ) Resultado da fixação do enxerto.

Foram confeccionados túneis ósseos nos ramos púbicos bilateralmente, a cerca de 1,5 cm lateral à sínfise púbica, com direção de anteroinferior para posterosuperior ( Fig. 2C ). Com auxílio de fios de alta resistência, o enxerto foi passado pelos túneis, configurando um trajeto em “U” em volta da sínfise púbica: no lado direito, no sentido crânio-caudal; no lado esquerdo, no sentido caudal-cranial ( Figs. 3A–C ). As extremidades do enxerto foram tracionadas posteriormente à sínfise com o objetivo de promover redução da diástase articular. Em seguida, procedeu-se à fixação do enxerto por meio de sutura direta no próprio tendão entre suas extremidades, sob tensão controlada com fios inabsorvíveis, fazendo uma configuração em oito ( Figs. 3D ). O excesso de enxerto foi ressecado ( Fig. 3E ). Ao término do procedimento, a estabilidade da articulação foi testada manualmente, confirmando o adequado tensionamento e fixação do enxerto ( Fig. 3F ).

No período pós-operatório, os pacientes foram submetidos a protocolos de reabilitação fisioterapêutica, com foco na mobilidade pélvica, recuperação funcional e controle da dor por meio de crioterapia e eletroestimulação neuromuscular. A descarga de peso foi permitida parcialmente com uso de andador ou duas muletas por 2 meses, seguida por mais 1 mês de proteção com uma muleta do lado mais comprometido funcionalmente.

A progressão do movimento articular foi facilitada pelo uso de equipamento de mobilização passiva contínua por 4 semanas, associado a cinesioterapia orientada.

A ativação do controle motor foi realizada por meio de exercícios de reforço de core apenas após 2 meses, com evolução gradual conforme tolerância clínica e resposta funcional do paciente.

Exercícios de pliometria em cama elástica foram instituídos apenas com 4 meses, com a volta à corrida após 5 meses e retorno ao esporte progressivo após 6 meses.

**Fig. 4:**
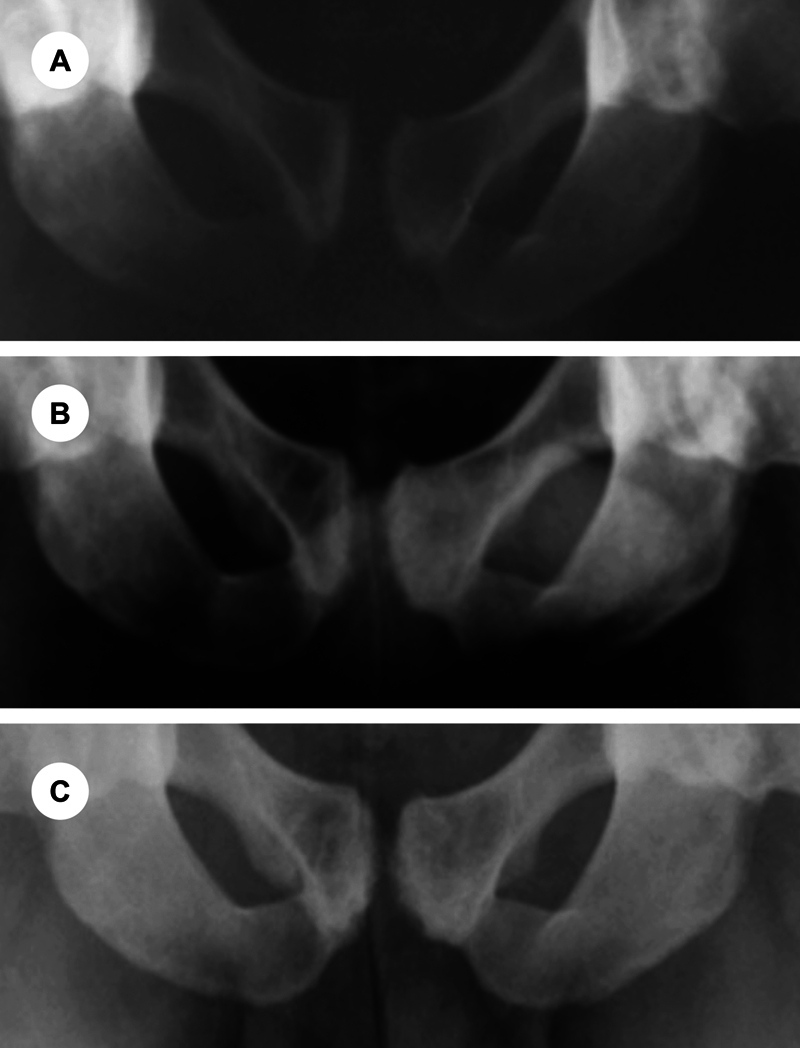
Radiografias seriadas da sínfise púbica: ( **A** ) Pré-operatório; ( **B** ) 6 meses de pós-operatório; ( **C** ) 42 meses de pós-operatório.

Os pacientes foram submetidos a avaliações seriadas com o objetivo de estimar a evolução clínica, funcional e estrutural da articulação da sínfise púbica, bem como da eficácia da reconstrução com enxerto autólogo, por meio da comparação dos parâmetros radiográficos, de ressonância magnética e da resposta clínica no período pré-operatório, 6 meses e 5 anos após o procedimento cirúrgico ( Fig. 4 ). A avaliação funcional foi realizada com o Escore de Harris Modificado para o Quadril. A intensidade da dor foi avaliada pela escala visual analógica, com melhora progressiva em ambos os casos como demonstrado na Tabela 1 .

**Tabela 1:** Escores de dor e funcionalidade dos pacientes no período pré-operatório e aos 6 meses e 5 anos da cirurgia

	Escala EVA (dor)		Escore de Harris modificado	
	Paciente 1	Paciente 2	Paciente 1	Paciente 2
Pré-operatório	10	8	45	58
6 meses	2	0	92	90
5 anos	0	0	90	97

**Abreviatura** : EVA, Escala Visual Analógica.

Como descrito, ambos os pacientes retornaram às atividades esportivas de alto rendimento sem restrições após 6 e 8 meses de pós-operatório, respectivamente, com melhora significativa da dor na escala analógica visual, após o protocolo de reabilitação descrito. Após 5 anos de seguimento, não foram observadas novas queixas relacionadas ao quadro prévio, dor sacro-ilíaca ou quaisquer complicações associadas.

## Discussão

As lesões da sínfise púbica representam um desafio significativo na prática ortopédica, especialmente quando associadas à instabilidade pélvica ou à falha de tratamentos conservadores e cirúrgicos prévios.

No primeiro caso descrito, o paciente havia sido submetido a uma ressecção da sínfise púbica, o que levou a uma piora da instabilidade pélvica, ocorrência relatada por Moore et al., 8 que relataram casos de mulheres com instabilidade após ressecção do púbis tratadas com artrose de sínfise púbica e de ambas articulações sacro-ilíacas, com bons resultados funcionais em 2 anos, apesar de lombalgia residual.

O segundo paciente apresentava mobilidade da sínfise púbica maior que 5 mm em radiografias de Flamingo, caracterizando instabilidade pélvica segundo os critérios radiológicos de Siegel et al. 7

Diversas técnicas cirúrgicas têm sido descritas na literatura para o manejo dessas lesões, incluindo artrodese com de placas de síntese com ou sem enxertos ósseos e reconstruções com materiais sintéticos. 9 10 11 12 13

Devido à raridade da lesão, todos os estudos são de séries de casos envolvendo de 1 a 8 casos, com taxas de sucesso variando de 67 a 100%. 14

A artrodese com placas ortogonais é a técnica mais descrita. Em 2 séries de atletas com 8 casos cada uma, revisadas por Williams et al., 9 Choi et al., 15 e Vitanzo et al. 16 os resultados de 52 meses de pós-operatório demonstraram 25% de complicações e retorno ao esporte em 83% dos casos.

Olucha-Puchol et al. 17 descrevem uma técnica cirúrgica em 2 casos de artrodese com uma placa e cerclagens metálicas, porém em mulheres não atletas e com idade superior a 50 anos.

A preocupação em nossos casos foi especialmente voltada para as consequências de uma artrodese em pacientes que planejavam ter ainda uma vida esportiva ativa e profissional, com possíveis sequelas para a biomecânica das articulações sacro-ilíacas e a coluna lombar. 9

Outro problema a prevenir seria a falha da síntese, que ocorreu em 43% de 148 casos traumáticos tratados com duas placas por Morris et al., 18 com realização de uma segunda cirurgia em 8% dos casos.

Investigações biomecânicas por meio da avalição de anéis pélvicos cadavéricos, demonstraram que a fixação fisiológica com sutura de fita (“tape suture”) proporciona estabilidade biomecânica adequada, sem exceder a mobilidade fisiológica de 2 mm da sínfise púbica, mesmo sob condições de carregamento cíclico vertical e horizontal de curta e longa duração. 19

Castropil et al. 20 compararam três técnicas para reconstrução da instabilidade esternoclavicular, articulação que tem biomecânica semelhante à da sínfise púbica: fixação intramedular, reforço com o tendão subclávio e reforço com o tendão semitendíneo em configuração em oito. Os resultados demonstraram que a técnica com tendão semitendíneo apresenta propriedades biomecânicas superiores às demais, sendo possível, portanto, aplicar o mesmo princípio para a articulação da sínfise púbica.

A técnica apresentada neste relato se propõe como uma alternativa biológica no tratamento da instabilidade crônica, uma vez que os enxertos autólogos de tendões isquiotibiais apresentam propriedades viscoelásticas que simulam a função ligamentar, sendo capazes de resistir às forças de cisalhamento e distração, ao mesmo tempo em que mantêm certo grau de elasticidade. 20

Assim como a técnica descrita por Arner et al., 5 na qual é realizada a fixação laparoscópica mediante o uso de âncoras e *tape suture* em configuração cruzada, a reconstrução da sínfise púbica utilizando enxerto de isquiotibiais busca restabelecer a estabilidade articular enquanto mantém certo grau de mobilidade fisiológica, mimetizando a função ligamentar e atendendo às demandas funcionais específicas do paciente.

A preservação da mobilidade da sínfise púbica é relevante em pacientes ativos ou atletas, pois os micromovimentos dessa articulação são essenciais para atividades que envolvem corrida, saltos e mudanças de direção. 12 A artrodese, por outro lado, elimina completamente essa mobilidade, alterando o padrão de movimento de forma a aumentar a sobrecarga em articulações adjacentes, como as sacro-ilíacas e a coluna lombar, comprometendo o desempenho esportivo. 4 13

Uma crítica ao uso do enxerto do tendão semitendíneo autólogo é o possível prejuízo à força de flexão do joelho. No entanto, este tendão é constantemente utilizado em reconstruções ligamentares no joelho devido à sua boa disponibilidade, resistência e baixo risco de complicações. Em ambos os casos os atletas realizaram, em pós-operatório tardio, testes isocinéticos com valores normais na relação flexores/ extensores de joelho, sem lesões de jarrete referidas no pós-operatório tardio. Uma alternativa seria a utilização de enxerto de banco de tecidos, para reconstrução biológica sem agredir área doadora. 6

A avaliação do Escore de Harris Modificado para o Quadril demonstrou uma boa evolução funcional, passando de mais de 30 pontos de melhora com apenas 6 meses de seguimento em ambos os casos.

A melhora da dor na avaliação da escala analógica após 6 meses e 5 anos de seguimento demonstrou que a técnica leva a uma melhora considerável na qualidade de vida destes pacientes.

Os resultados sugerem que essa abordagem cirúrgica pode contribuir para o aperfeiçoamento das opções terapêuticas em pacientes com instabilidade anterior da sínfise púbica e auxiliar na validação de uma técnica cirúrgica não descrita na literatura, com potencial de restaurar a estabilidade e fisiológica da sínfise púbica, mesmo a médio prazo (5 anos).

No entanto, este estudo inclui apenas dois pacientes, o que reflete a escassez de dados disponíveis na literatura, que se limita, em grande parte, a pequenas séries de casos devido à raridade da patologia. Isso reforça a necessidade de futuros estudos, com amostras maiores, preferencialmente de séries prospectivas caso-controle e com seguimento a longo prazo.

## Conclusão

Foi observada melhora dos sintomas clínicos e retorno funcional às atividades físicas, com manutenção da estabilidade pélvica radiológica durante o seguimento de 5 anos. A técnica de reconstrução com enxerto autólogo do tendão semitendíneo demonstrou ser segura, com baixa morbidade e com bons resultados funcionais segundo o Escore de Harris Modificado para o Quadril nestes dois atletas de futebol profissional. Entretanto, estudos com maior casuística são necessários para reproduzir estes resultados.
